# Viral tumor antigen expression is no longer required in radiation-resistant subpopulation of JCV induced mouse medulloblastoma cells

**DOI:** 10.18632/genesandcancer.174

**Published:** 2018-03

**Authors:** Martina Donadoni, Rahsan Sariyer, Hassen Wollebo, Anna Bellizzi, Ilker Kudret Sariyer

**Affiliations:** ^1^ Department of Neuroscience and Center for Neurovirology, Temple University Lewis Katz School of Medicine, Philadelphia, PA, USA

**Keywords:** JC virus, PML, cancer, medulloblastoma, viral oncogene

## Abstract

The human neurotropic polyomavirus JC, JC virus (JCV), infects the majority of human population during early childhood and establishes a latent/persistent infection for the rest of the life. JCV is the etiologic agent of the fatal demyelinating disease of the central nervous system, progressive multifocal leukoencephalopathy (PML) that is seen primarily in immunocompromised individuals. In addition to the PML, JCV has also been shown to transform cells in culture systems and cause a variety of tumors in experimental animals. Moreover, JCV genomic DNA and tumor antigen expression have been shown in a variety of human tumors with CNS origin. Similar to all polyomaviruses, JCV encodes for several tumor antigens from a single transcript of early coding region via alternative splicing. There is little known regarding the characteristics of JCV induced tumors and impact of DNA damage induced by radiation on viral tumor antigen expression and growth of these cells. Here we analyzed the possible impact of ionizing radiation on transformed phenotype and tumor antigen expression by utilizing a mouse medulloblastoma cell line (BSB8) obtained from a mouse transgenic for JCV tumor antigens. Our results suggest that a small subset of BSB8 cells survives and shows radiation resistance. Further analysis of the transformed phenotype of radiation resistant BSB8 cells (BSB8-RR) have revealed that they are capable of forming significantly higher numbers and sizes of colonies under anchorage dependent and independent conditions with reduced viral tumor antigen expression. Moreover, BSB8-RR cells show an increased rate of double-strand DNA break repair by homologous recombination (HR). More interestingly, knockout studies of JCV tumor antigens by utilizing CRISPR/Cas9 gene editing reveal that unlike parental BSB8 cells, BSB8-RR cells are no longer required the expression of viral tumor antigens in order to maintain transformed phenotype.

## INTRODUCTION

JC virus (JCV) is a human polyomavirus, which infects the majority of human population during early childhood, forms a latent/persistent infection for the rest of the life, and reactivates in individuals mostly under immunosuppressive conditions leading to the development of progressive multifocal leukoencephalopathy (PML). JC virus genomic DNA can be detected in serum and urine of immunocompetent individuals that suggests the presence of a low level viral replication leading to viral persistency in healthy subjects [1, 2, 3]. Beside its role in the development of PML, JC virus has also been associated with various tumors in laboratory animals and humans. Similar to the simian polyomavirus 40 (SV40), JC virus also shows ability to transform primary cells in vitro [4]. JCV-transformed primary human cells express viral transforming antigens and exhibit transformed phenotype [5, 6].

On the other hand, inoculation of JCV into experimental animals, including mice, hamster, and primates results in tumor development rather than lytic viral replication. Intracerebral inoculation of JCV PML strain into Syrian hamsters leads to the development of glial and neuronal origin tumors including glioblastomas, neuroblastomas, and medulloblastomas [7, 8]. JCV has also been shown to be tumorigenic in nonhuman primates [9, 10]. Mice lines transgenic for JCV early coding region encoding for viral tumor antigens under the control of viral promoter were also created. Interestingly, viral promoter activity was attributed to the neuronal cells with the formation of different tumors that derived from neural origin in these transgenic mice models [11, 12, 13].

JCV genomic sequences and viral proteins have also been detected and reported in variety of human tumors. Sporadic development of human tumors with CNS origin, such as oligodendroglioma, astrocytomas, and neuroblastomas were reported in PML patients [14, 15, 16]. Expression of viral tumor antigens was observed in the absence of productive lytic infection in PML patients. Expression of the JCV large T antigen and presence of JCV genome have also been detected in human brain tumors in the absence of PML lesions [17, 18, 19, 20]. Such findings provided evidence for a possible association of JCV for the formation of human tumors with CNS origin. In fact, according to Del Valle et al, 2001 and 2002 [19, 20], JCV early gene sequences were detected in 62.5% of oligoastrocytomas, 83.3% of ependymomas, 80% of pilocytic astrocytomas, 57.1% of oligodendrogliomas, 76.9% of astrocytomas, and 66% of anaplastic oligodendrogliomas.

The oncogenic potential of JCV is strongly associated with the expression of viral tumor antigens. Several line of evidence suggests that JCV-mediated cellular transformation relies on the sequestration and suppression of the tumor suppressor proteins, p53 and the pRb family, by the viral large T antigen [21, 22, 23]. JCV large T antigen can also interact with other cellular proteins such as insulin receptor substrate 1 (IRS-1), β-catenin, neurofibromatosis type 2 gene product, and antiapoptotic protein survivin which are also implicated in pathways associated with cellular transformation, [24, 25, 26, 27, 28]. We have previously showed that downregulation of JCV tumor antigen expression in BSB8 cells, a cell line that originated from a medulloblastoma developed in a transgenic mouse expressing the JCV-early region, and in HJC-2 cells, a cell line obtained from a glioblastoma induced by intracranial injection of JCV in newborn hamsters, results in growth arrest and induction of apoptosis [6]. These observations suggest that the fate of cells transformed by JCV might be indeed depended on viral tumor antigen expression. However, the possible impact of secondary insults, such as ionizing radiation, on viral gene expression and growth of tumor cells transformed by JCV has not been reported.

Here we investigated the effects of ionizing radiation on JCV tumor antigen expression and viability of BSB8 cells. Interestingly, although radiation induced apoptosis in the majority of BSB8 cells, a small subset of cells showed radiation resistance. Further analysis of radiation resistant subpopulation of BSB8 cells (BSB8-RR) revealed that these cells expressed reduced levels of viral tumor antigens, were no longer sensitive to repeated insults of radiation, had an increase in cell-cycle, and were able to repair double-strand breaks with a better efficiency than parental cells. Moreover, knockout of JCV tumor antigens from BSB8-RR cells by excision mediated by CRISPR/Cas9 gene editing suggest that viral tumor antigen expression was no longer required for the growth and colonization of the BSB8-RR cells. These results may have implications for the involvement of JCV tumor antigens in early stages of human cancers and suggest a possible mechanism of radiation resistance.

## RESULTS

### Effect of radiation on mouse medulloblastoma cells induced by JCV tumor antigens

**Figure 1:**
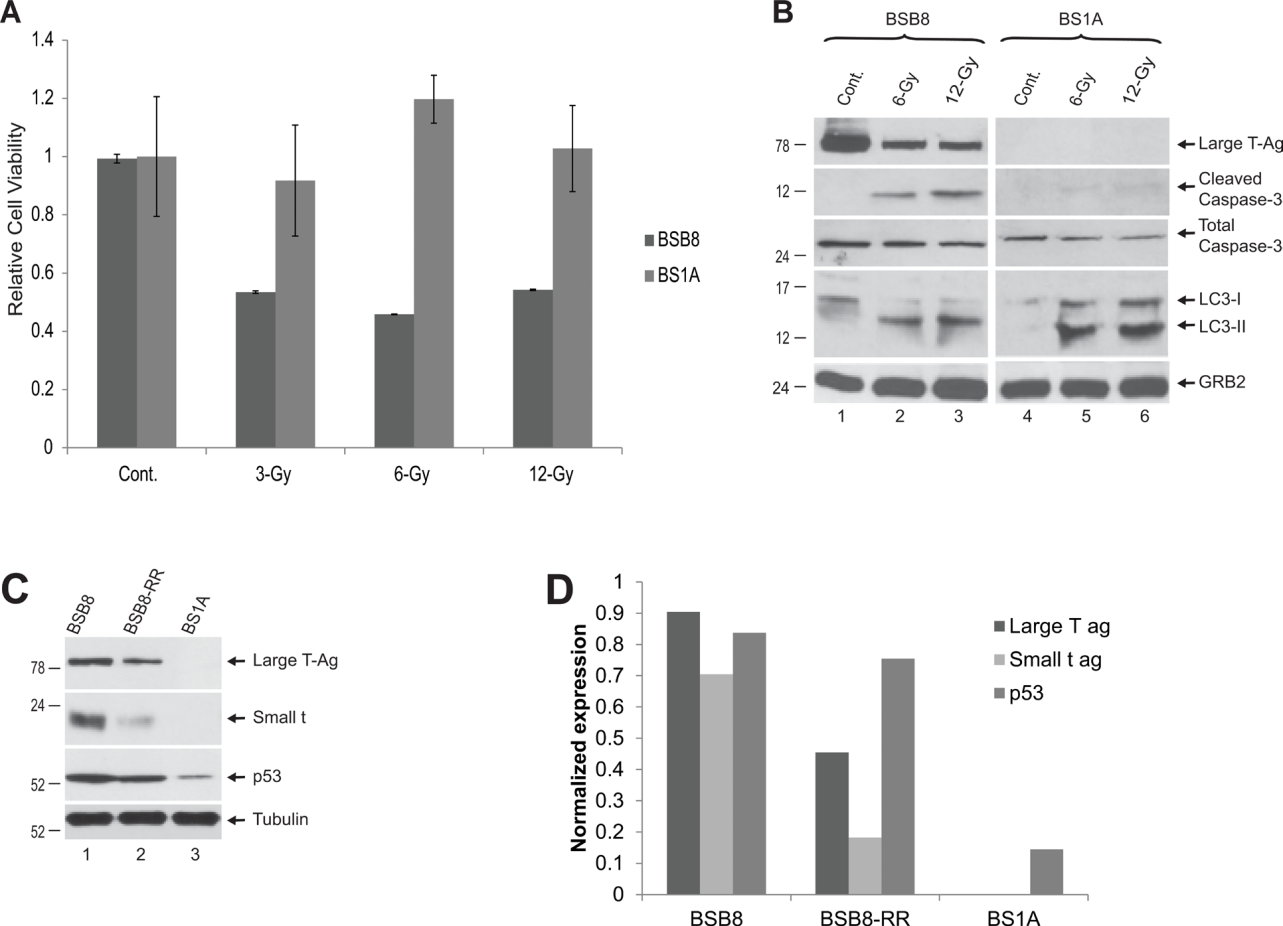
Effect of radiation on apoptosis and autophagy **A.** MTT assay of BS1A and BSB8 cells after treatment with ionizing radiation. **B.** Expression of proteins involved in apoptosis and autophagy in BS1A and BSB8 cells after radiation treatment. **C.** Western blot analysis for Large T-Ag, small-t, and p53 in BSB8, BSB8-RR, and BS1A cells. **D.** Band intensities of Large T antigen, small t antigen, and p53 from Western blot analysis in panel C were normalized to tubulin and shown as bar graph.

JC virus can transform cells in culture systems and cause a variety of tumors in experimental animals. Cell lines from a medulloblastoma induced in a mouse model transgenic for JCV tumor antigens were generated previously [11]. Among these cell lines, we utilized BSB8 cells, which express viral tumor antigens and shows transformed phenotype and BS1A cells, which were immortal but did not express viral tumor antigens with no obvious transformed phenotype. BSB8 and BS1A cells were first grown to full confluency in tissue culture dishes. Cells were exposed to various dosages of ionizing radiation (3-6-12 Gy) and analyzed by MTT cell viability assay at 72h post-radiation. As shown in Figure 1A, radiation had no impact on BS1A cell viability due to the contact inhibition of their growth at full confluency. On the other hand, radiation caused a significant reduction in cellular viability of BSB8 cells at 3-Gy and did not further alter cellular viability loss at 6 and 12-Gy dosages. In parallel, whole cell protein extracts were also obtained from BSB8 and BS1A cells exposed to ionizing radiation at 6 and 12-Gy dosages and analyzed by Western blotting at 72hrs post-radiation. As shown in Figure 1B, radiation caused a significant decrease in JCV large T-ag expression at 6-Gy with no further change at 12-Gy. Interestingly, both apoptosis and autophagy were induced in BSB8 cells as evidenced by cleaved caspase-3 activation and LC3-II conversion, respectively (Figure 1B, compare lane 1 with lanes 2 and 3). On the other hand, BS1A cells did not show any large T-ag expression or cleaved caspase-3 activation although LC3-II conversion was induced by radiation (Figure 1B, compare lane 4 with lanes 5 and 6). These results suggest that ionizing radiation induces autophagy in both BSB8 and BS1A cells but apoptosis is only induced in BSB8 cells. The observed impact of radiation on BSB8 cell viability and large T-ag expression were not altered by increased radiation dosages suggesting that a subset of these cells were possibly resistant to the apoptosis induced by radiation. In order to test this, BSB8 cells were irradiated at 6-Gy and nearly 80% of cells were lost due to the extensive apoptosis by 72h post radiation. The remaining radiation resistant sub-population of BSB8 cells was further cultured for two weeks and called “BSB8-RR”. As shown in Figure 1C-1D, BSB8-RR cells retained the low levels of large T-ag and small-t-ag expressions with no significant difference in p53 levels as compared to original BSB8 cells. On the other hand, basal p53 expression levels were significantly lower in BS1A cells than BSB8 and BSB8-RR cells with no sign of JCV tumor antigen expression as expected.

### BSB8-RR cells are resistant to repeated radiation insults

**Figure 2:**
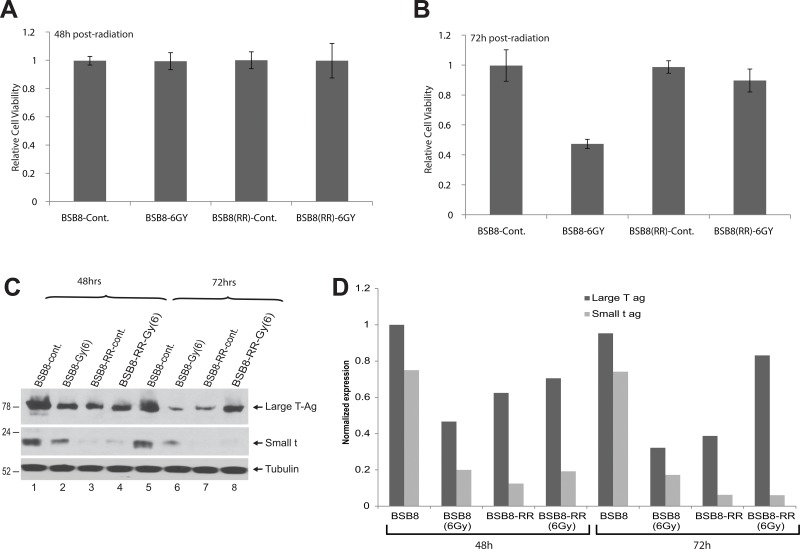
BSB8-RR cells show resistance to secondary radiation **A.** MTT assay on BSB8 and BSB8-RR control or irradiated with dose of 6-Gy at forty-eight hours after radiation. **B.** MTT assay on BSB8 and BSB8-RR control or irradiated with dose of 6-Gy at seventy-two hours after radiation. **C.** Western blot analysis of Large T-Ag and small-t in control and irradiated BSB8 and BSB8-RR cells at 48 and 72hrs post-radiation. **D.** Band intensities of Large T antigen and small t antigen from Western blot analysis in panel C were normalized to tubulin and shown as bar graph.

To further investigate the role of JCV tumor antigen expression in resistance to the radiation, BSB8 and BSB8-RR cells were irradiated at 6-Gy and cellular viability was analyzed by MTT assay at 48h and 72h post-radiation. While cellular viabilities of both BSB8 and BSB8-RR cells were not altered at 48h post-radiation, there was a significant decrease in BSB8 viability at 72h (Figure 2A and B). Interestingly, unlike BSB8 cells, BSB8-RR cells did not show any significant alteration in cellular viabilities even after 72h post-radiation (Figure 2B, compare lane 4 with 2) suggesting that they were resistant to cell death induced by secondary radiation. In parallel, whole cell extracts were also prepared and processed by Western blotting for the detection and analysis of viral tumor antigen expression from the same set of studies. As shown in Figure 2C and 2D, viral large T-ag and small t-ag expressions were dramatically reduced in BSB8 cells at 48 and 72h post-radiation. On the other hand, while there was no significant alteration at 48h, large T-ag expression was induced by secondary radiation in BSB8-RR cells at 72h post-radiation (Figure 2C and 2D, compare lane 3 with 4 and 7 with 8). Interestingly, unlike large T-ag expression, small t-ag levels were not altered by secondary radiation in BSB8-RR cells. These data suggest that the alterations in the expression levels of tumor antigens before and after radiations in original BSB8 cells and BSB8-RR cells may have a role in the resistance to the radiation.

### BSB8-RR cells show increased cell-cycle rates and grow more aggressively than original BSB8 cells under anchorage dependent and independent conditions

**Figure 3:**
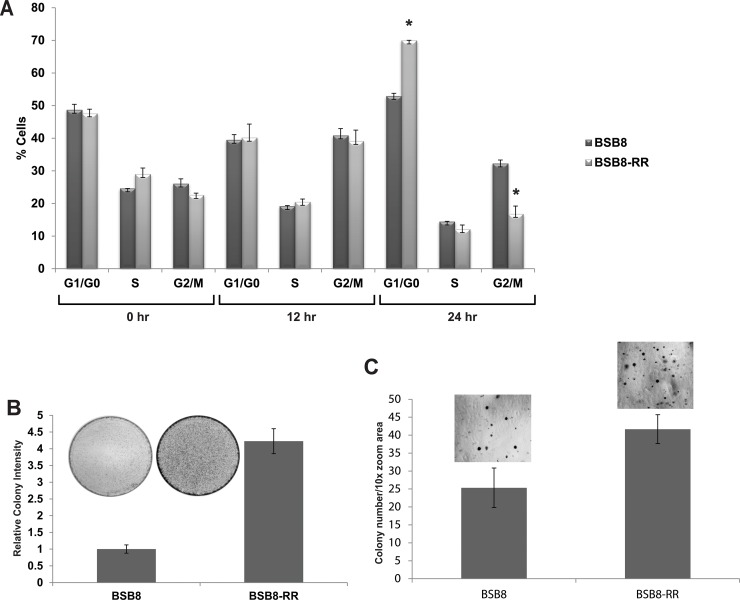
Growth characteristics of BSB8 and BSB8-RR cells **A.** Cell cycle analysis of BSB8 and BSB8-RR cells at three time points after FBS deprivation. **B.** BSB8-RR cells form increased number of colonies under anchorage-dependent conditions. Colony formation assay after transfection with pcDNA3.1 and selection with G418 treatments. **C.** BSB8-RR cells form increased number and size of colonies under anchorage independent conditions. Soft agar growth after transfection with pcDNA3.1 and selection with G418.

As shown above, BSB8-RR cells show alterations in viral tumor antigen expression and are resistant to the secondary radiation (Figures 1 and 2). Next we asked if there were also any alterations in their growth characteristics and tumorigenic capacity compared to original BSB8 cells. In order to investigate and compare growth rates, BSB8 and BSB8-RR cells were first serum-starved for 24 hours. Starvation was released by adding 10% fetal bovine serum and cells were harvested at 0h-12h-24h post-release from starvation. Cells were treated with propidium iodide and cell-cycle analyses were performed using flow cytometry. As shown in Figure 3A, both BSB8 and BSB8-RR cells had a synchronized G1/G0 stage at 0h (24h post-starvation) as expected. Interestingly, BSB8-RR cells showed a significantly higher percentage of cells at G1/G0 and lower percentage of cells at G2/M after 24h post-starvation release suggesting that initiation and completion of cell cycle of BSB8-RR cell were most likely faster than the original BSB8 cells. Following cell cycle analysis, growth characteristics and tumorigenesis of BSB8 and BSB8-RR cells were also analyzed by colony formation assay and soft agar growth analyses under anchorage dependent and independent conditions, respectively. As shown in Figure 3B, BSB8-RR cells form significantly higher number of colonies under anchorage-dependent conditions than BSB8 cells as evident by the increased intensities of the colonies formed. Furthermore, BSB8-RR cells also formed significantly higher number of colonies in soft agar under anchorage-independent conditions (Figure 3C) suggesting that they acquire a greater tumorigenicity.

### BSB8-RR cells show reduced NHEJ but increased homologous recombination DNA repair

**Figure 4:**
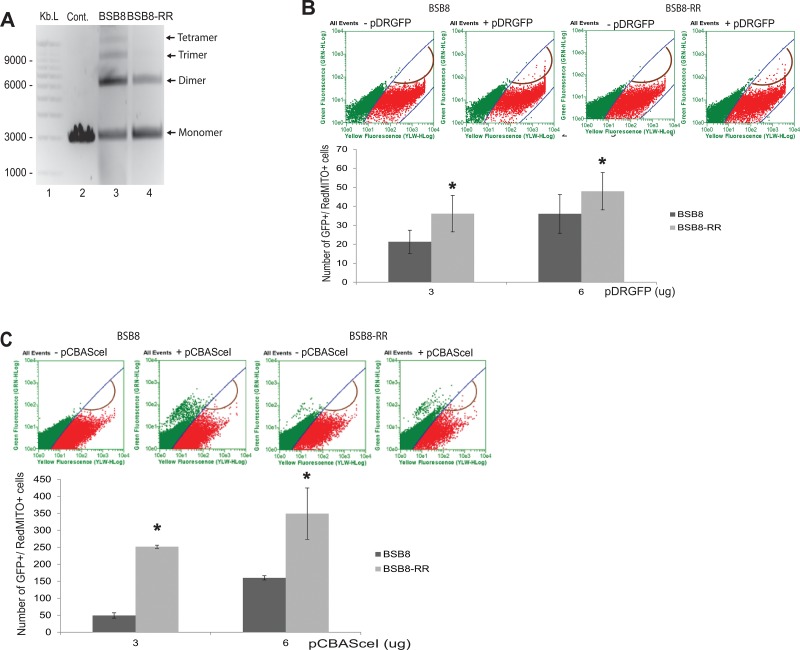
DNA repair properties of BSB8 and BSB8-RR cells **A.** Non-homologous end joining (NHEJ) activity in nuclear extracts from BSB8 and BSB8-RR cells. **B.** Representative scatter plots of untransfected cells, transfected BSB8 and BSB8-RR with pCBASceI and pDsRedl-Mito but not pDRGFP and BSB8 and BSB8-RR transfected with pCBASceI + pDsRedl-Mito + pDRGFP. Quantification of green positive cells after transfection with 3 ug and 6 ug of pDRGFP is represented as bar graph. **C.** Representative scatter plots of transfected BSB8 and BSB8-RR with pDsRedl-Mito and BSB8 and BSB8-RR transfected with pCBASceI + pDsRedl-Mito. Quantification of green positive cells after transfection with 3 ug and 6ug of pCBASceI is represented as bar graph.

Ionizing radiation holds its application by causing extensive double-strand DNA breaks in cancer cells leading to the activation of apoptosis and cell death. Upon radiation the fate of cancer cells is truly depended on how fast and efficient they can repair the DNA damage induced by radiation. As shown above, BSB8-RR cells were originated from the subpopulation of BSB8 cells after radiation and they were resistant to the secondary radiation. Moreover, they showed increased cell-cycle rates and grew more aggressively than original BSB8 cells. We next analyzed and evaluated double-strand break DNA repair efficiencies of BSB8 and BSB8-RR cells. Double-strand DNA breaks can be repaired by non-homologous DNA end joining (NHEJ) or homologous recombination (HR). Regarding the NHEJ assay, nuclear extracts were prepared from BSB8 and BSB8-RR cells and analyzed for NHEJ activity as described in materials and methods. As shown in Figure 4A, nuclear extracts from BSB8-RR cells had a lower NHEJ activity than the nuclear extracts from the original BSB8 cells (compare lane 4 with 3) as evident from the formation of dimer, trimer, and tetramer forms of the plasmid DNA. In addition, DNA repair activity of BSB8 and BSB8-RR cells was also analyzed by homologous recombination assay as described previously [29]. Cells were transiently transfected with pCBASceI, pDsRedl-Mito, and increasing amounts of pDRGFP plasmids by electroporation. Cells were harvested and analyzed by flow cytometry for the percentages of green and red fluorescein cells at 72h post-transfections. The efficiency in repairing double-strand DNA breaks using homologous recombination was established as the number of cells positive for both green and red fluorescence normalized to the cells positive only for red fluorescence. As shown in Figure 4B, number of GFP and Red-mito expressing cells were significantly higher in BSB8-RR cells than BSB8 cells. Since these experiments required transient transfection of three plasmids with considerably low co-transfection efficiencies, BSB8 and BSB8-RR cells were also transfected with pDRGFP plasmid and stable single-cell clones were generated. To confirm transient transfection results, BSB8 and BSB8-RR cells with stable pDRGFP expression were transfected with pDsRedl-Mito alone or in combination with increasing concentrations of pCBASceI plasmid. Cells were harvested and analyzed by flow cytometry for the number of green and red fluorescein cells at 96h post-transfections. As shown in Figure 4C, consistent with transient transfection studies, number of GFP and red positive cells were significantly higher in BSB8-RR cells than original BSB8 cells suggesting that BSB8-RR cells possess an increased rate of double-strand DNA repair by homologous recombination.

### BSB8-RR cells do no longer require JCV tumor antigen expression for the maintenance of the transformed phenotype

**Figure 5:**
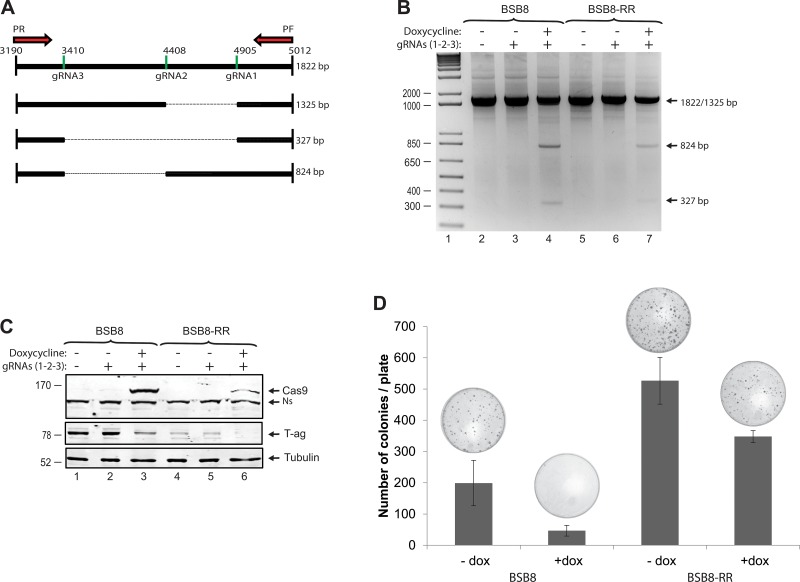
BSB8-RR cells do not require expression of viral tumor antigens for the maintenance of transformed phenotype **A.** Schematic representation of JCV tumor antigen genes and position of gRNAs with expected size of amplification products upon cleavage by CRISPR/Cas9. **B.** PCR analysis of genomic DNA isolated from BSB8 and BSB8-RR cells stable for Cas9 expression upon induction with doxycycline and transduction with lentiviruses encoding gRNAs 1,2,3. C. Western blot analysis of whole cell protein lysates prepared from the same set of experiments presented in panel B for the detection of Cas9 and large T-ag. D. Colony formation assay and quantification of number of colonies in BSB8 and BSB8-RR cells after Cas9 induction with doxycycline.

The BSB8 cells were originally obtained from a mouse medulloblastoma developed in a mouse transgenic for JCV tumor antigens [11]. They show transformed phenotype and express viral early gene products (tumor antigens). Theoretically, their transformed phenotype and viabilities are depended on expression of viral tumor antigens. Next, we analyzed and compared the impact of JCV tumor antigen expression on transformed phenotype of BSB8 and BSB8-RR cells by CRISPR/Cas9 gene editing strategy. Sub-cell lines of both BSB8 and BSB8-RR cells were first generated for doxycycline inducible expression of Cas9. Cells were then transduced with the combination of lentiviruses encoding three gRNAs targeting various regions within the viral early coding sequence (Figure 5A) as described previously [30]. Cells were either treated or untreated with doxycycline for the induction of Cas9 expression and cellular DNA were extracted for excision by PCR utilizing a pair of primers spanning the all gRNA target sites. As shown in Figure 5B, the excision products of 327bp and 824bp were amplified for gRNAs 1/3 and gRNAs 2/3, respectively, in both BSB8 and BSB8-RR cells. In parallel to the DNA samples, whole cell protein extracts were also collected from the same set of experiments. Expression of Cas9 and down-regulation of JCV tumor antigen expression were also analyzed and confirmed by Western blotting (Figure 5C). Finally, effect of tumor antigen knockout on growth and transformed phenotype of BSB8 and BSB8-RR cells were analyzed by colony formation assay. As shown in Figure 5D, as expected, BSB8-RR cells were able to form much higher numbers of colonies than BSB8 cells. Interestingly, BSB8 cells with doxycycline treatment leading tumor antigen knockout showed a dramatic reduction in the number of colonies compared to control BSB8 cells, suggesting that the growth and colony forming ability of BSB8 cells were truly depended on JCV tumor antigen expression. On the other hand, number of colonies formed by BSB8-RR cells with tumor antigen knockout was slightly reduced compared to the control cells (compare lane 3 with 4), but it was still significantly higher than original BSB8 cells with tumor antigen expression (compare lanes 4 and 1) suggesting that JCV tumor antigen expression was no longer needed for the maintenance of the transformed phenotype in BSB8-RR cells.

## DISCUSSION

JCV infection in experimental animals, including hamsters, nonhuman primates, and mice results in cellular transformation and tumor development rather than lytic viral replication suggesting that viral early promoter is activated for the expression of tumor antigens leading to genome instability and inactivation of oncosuppressor proteins that eventually may lead to tumorigenesis [31]. Radiotherapy is commonly used as a part of cancer therapy in patients with many different forms of localized tumors before and after surgical operations. Regarding postsurgical application, radiation is commonly used to eliminate any remaining tumor cells at the side of surgery. Unfortunately, some small subset of tumor cells may become resistant to radiation and be responsible for the tumor recurrence. In order to gain insight into the effects of radiation on tumor cell growth, we utilized BSB8 cells derived from a medulloblastoma developed in a mouse transgenic for JCV [11]. As expected, radiation of BSB8 cells resulted in induction of autophagy along with apoptosis in the majority of the cells in the culture plate. Interestingly, a small subset of cells survived, become resistance to repeated dosages of radiation with reduced levels of viral tumor antigen expression. Since the JCV tumor antigens were the main driver of transformation in these cells, the reduction in its expression levels in BSB8-RR cells was not anticipated. JCV large tumor antigen is a phosphoprotein, which interacts with two key tumor suppressor proteins that regulate cell cycle progression, pRb and p53 [32, 33]. Possible alteration of its posttranslational modification, interaction with p53 and Rb proteins, and binding to viral promoter in BSB8-RR compared to original BSB8 cells remains to be further characterized.

Our results have revealed several interesting features of BSB8-RR cells that include an increase in cell cycle rate and number of colonies formed under anchorage-dependent and -independent conditions suggesting that adaptation to the radiation led to the more aggressive tumor phenotype of these cells. Further analysis for the possible mechanism of this adaptation and change in growth phenotype of the BSB8-RR cells revealed that they possessed an increase rate of double-strand DNA break repair. These results are in agreement with previous observations indicating that radiation-resistant cancer cells can gain adaptation to repair double-strand breaks induced by radiation [34, 35, 36]. On the other hand, only a small subset of cells shows resistance to the radiation while the majority of the cells are highly responsive. Although cancers become more heterogeneous during the course of tumor development [37], factors contributing to the radiation resistance in a small subset of tumor cells and role of viral tumor antigens in this progress remains to be determined.

Whether JCV is involved in tumors in human have been debated since the virus first isolated. It is without any concern that JCV can transform primary cells in vitro and lead to formation of various tumors in experimental animals [7, 8, 9, 10, 11, 12, 13]. Moreover, viral tumor antigens and genomic sequences were detected in human neuronal origin tumors, including glioblastoma, oligodendroglioma, and medulloblastoma by our group and various labs across the globe [14, 15, 16, 17, 18, 19, 20]. Among all polyomaviruses, merkel cell polyomavirus (MCPyV) is the only polyomavirus discovered as the underlying etiology of human merkel cell carcinoma, an aggressive form of skin cancer [38, 39]. MCPyV and JCV show very similar genomic organization with conserved homologies in viral tumor antigen sequences. Recent studies indicate that viral tumor antigen expression is required for the growth of merkel cell carcinoma cells [40, 41]. Unlike MCPvY, our results suggest for the first time that the growth of tumor cells induced by JCV does no longer required the expression of tumor antigens in order to sustain their transformed phenotype. This unique observation may have implications for possible involvement of JCV in the formation of human tumors with CNS origin. One possibility is that the expression of viral tumor antigens in the absence of viral replication may initiate the cellular transformation process in glial cells persistently infected with the virus. Due to the additional insults, such as UV irradiation and DNA damaging agents/chemicals, tumor cells may gradually gain adaptation and tolerate the absence of viral tumor antigen expression for their growth. This type of phenomena of tumorigenesis can be explained by “hit-and-run” strategy as proposed previously for JCV [42]. Likewise, “hit-and-run” mechanism has also been proposed in the transformation initiated by the viral oncoproteins expressed by adenovirus [43], Hepatitis B virus [44], and more recently for the Beta HPV38 virus [45]. In line with these observations, our results support the concept that tumor antigens expressed by JCV act at the initial stages of the cellular transformation and secondary insults, such as radiation, may relief the cells for the need of their expression.

In conclusion, our results suggest that radiation triggers a cascade of events in tumor cells induced by JCV leading to elimination of their dependency for the expression of viral tumor antigens. These observations may suggest possible involvement of JCV in human tumors and open a new avenue of research to better understand and characterize the role of JCV in human tumors with CNS origin.

## MATERIAL AND METHODS

### Cell lines and cultures

The mouse medulloblastoma cell line expressing JCV tumor antigens (BSB8) and BS1A cells were previously described [11]. BSB8-radiation resistant (BSB8-RR) cells were obtained from radiation resistant subpopulation of BSB8 cells. BS1A, BSB8 and BSB8-RR cells were grown in Dulbecco's Modified Eagle's Medium (DMEM) containing 5% heat-inactivated FBS and 100μg/ml penicillin/streptomycin. All the cells were maintained at 37°C in a humidified atmosphere with 7% CO2.

### MTT assay for cell viability

BSB8, BSB8-RR or BS1A cells were plated in a concentration of 3 × 10^5^ cells / well in 6-well tissue culture plates and irradiated with different amount of ionizing radiation (3-Gy, 6-Gy or 12-Gy). 48 hours after treatment, cells were incubated with 1 ml of MTT (3-(4 5-dimethylthiazol-2-yl)-2 5-diphenyltetrazolium bromide), working solution (0.5 mg/ml) for 2 hours at 37°C. The converted insoluble purple formazan was solubilized with 1 ml of acidic isopropanol (0.004 M HCl in isopropanol). Absorbance of the converted formazan was measured at a wavelength of 570 nm with a background subtraction at 650 nm.

### SDS-PAGE and western blotting

Whole cell protein extracts were prepared from BSB8, BSB8-RR, and BS1A cells with TNN lysis buffer (40 mM Tris-HCl pH 7.4, 150 mM NaCl, 1 mM DTT, 1 mM EDTA, 1% NP40, and 1% protease inhibitors cocktail). Protein concentrations were quantified using Bradford reagent. Protein extracts were then heated to 95°C for 5 min and resolved through sodium dodecyl sulfate polyacrylamide gel electrophoresis (SDS-PAGE). Then they were transferred to nitrocellulose membranes in a transfer buffer containing 25 mM Tris base (pH 7.4), 200 mM glycine, and 20% methanol. After transfer, membranes were blocked for 30 min at room temperature with 10% non-fat dry milk in 1X phosphate-buffered saline containing 0.1% Tween-20 (PBST). Membranes were then incubated with primary antibodies overnight at 4°C. The membranes were subsequently washed three times with PBST and incubated with secondary antibodies at a 1:5000 dilution for 1 h at room temperature. After secondary antibodies incubation, membranes were visualized with an Odyssey CLx Imaging System (LI-COR).

### Cell cycle analysis

BSB8 and BSB8-RR cells were harvested at three different time point: 0h, 12h, 24h. Cells were washed with PBS, re-suspended in 1 ml of PBS and added drop-wise to 4 ml of 88% ethanol for the fixation in a final concentration of 70% ethanol. Cells were then washed with PBS, re-suspended in 300 μl of PBS and incubated with 10 μg/ml of propidium iodide and 100ug/ml solution of RNase A at 37°C for 30 minutes at dark. After incubation, cells were cooled at 4°C and data were acquired using a Guava EasyCyte Mini flow cytometer (Guava Technologies).

### Colony formation assay

To assess tumorigenesis, BSB8 and BSB8-RR cells either control or clones with inducible expression of Cas9 were plated in 100 mm tissue culture dishes. BSB8-Cas9 and BSB8-RR-Cas9 cells were transduced with lentiviral constructs encoding gRNAs 1, 2, and 3 for JCV early region and plated for colony formation assays in the presence or absence of doxycycline. Cells were kept in DMEM complete medium for two to three weeks till colonies were formed and visually observable. Colonies were fixed and stained with 0.1% crystal violet in 20% (vol/vol) aqueous methanol solution.

### Soft agar growth

BSB8 and BSB8-RR cells were transfected with pcDNA3.1 (+) and after 24 hours harvested and seeded in 60-mm dishes containing 2 mL of a 0.3% agarose suspension in DMEM complete medium with G418. Plates were then incubated at 37°C with for three to four weeks. Colonies were fixed and stained with 0.1% crystal violet in 20% (vol/vol) aqueous methanol solution and counted.

### Nonhomologous end joining assay for double strand DNA break repair

The nonhomologous end joining (NHEJ) assay was performed by a modification of the method of Baumann and West, 1998 as previously described [46]. Nuclear protein extracts from BSB8 and BSB8-RR cells were prepared as follows. Cells were harvested, washed with ice-cold PBS and re-suspended in ice-cold hypotonic lysis buffer A (10 mM HEPES pH 7.9, 10 mM KCl, 1.5 mM MgCl_2_, 1 mM DTT and protease inhibitors) and left on ice for 15 minutes. Integrity of cellular membrane was disrupted by adding 50 μl 10% NP-40 solution per 500μl cell suspension and gently vortexing for 10 seconds. Cell nucleus was pelleted by centrifugation at 1,800 rpm for 5 minutes and the supernatants were aliquoted as cytoplasmic extracts. Nuclear pellets were re-suspended in ice-cold PBS and washed to remove any remaining cytoplasmic proteins. Nuclear pellets then were re-suspended in ice-cold hypertonic nuclear lysis buffer C (20 mM HEPES pH 7.9, 0.6 M KCl, 1.5 mM MgCl_2_, 0.2 mM EDTA, 25% (v/v) glycerol, 1 mM DTT, 0.5 mM PMSF and protease inhibitors), rotated 45 minutes at 4°C, and the nuclear lysates were obtained by centrifugation at 15k rpm for 15 minutes. Supernatants were aliquoted as nuclear extracts and dialyzed overnight against a dialyzing buffer consist of 25 mM Tris HCl (pH 7.5), l mM EDTA, 10% glycerol (vol/vol) and protease inhibitors. The NHEJ assay was performed as follows. pBlueScript(KS+) plasmid was linearized by restriction endonuclease digestion in order to obtain a four nucleotides 5′ overhang using Bam HI, and 3′- blunt-end using EcoR V restriction enzymes. The resulting 3 Kb linear DNA was used as DNA substrate for the end-joining reactions. Reactions were set up in 25 mM Tris OAc (pH 7.5), 100 mM KOAc, 10 mM MgOAc, 1 mM DTT, 2 mM ATP and 200 mM dNTPs. In all reaction, total of 50 μg nuclear extract from BSB8 or BSB8-RR cell were utilized. The reactions were incubated for 5 min at 37°C before addition of 400 ng of DNA substrate followed by one hour incubation at 37°C. DNA products were de-proteinized by treatment with proteinase K and analyzed by electrophoresis on a 0.7% agarose gel.

### Homologous recombination assay for double strand DNA break repair

The homologous recombination (HR) events were analyzed as previously described (Pierce et al., 1999) with some modifications. For the transient transfection, BSB8 and BSB8-RR cells were electroporated with expression plasmids pDRGFP (recombination substrate), pCBASceI (encoding I-SceI endonuclease), and pDsRedl-Mito (Clontech, Paolo Alto, CA). The expression of I-SceI leads to a double strand break (DSB) in the specific restriction site within the pDRGFP cassette and HR events in the cells can restore the functional GFP expression. The co-transfection with pDsRedl-Mito, a plasmid that encodes for a red fluorescent protein with a mitochondrial localization, was used to calculate the efficiency of the transfection. Seventy-two hours after electroporation, cells were harvested and GFP and RFP expressions were detected and quantified using Guava EasyCyte Mini flow cytometer (Guava Technologies). Sub-cell lines of BSB8 and BSB8-RR cells were also obtained for stable expression of DRGFP as follow: BSB8 and BSB8-RR cells were electroporated with pDRGFP plasmid. Twenty-four hours after electroporation, cells were harvested and re-plated in 100-mm dishes with complete DMEM media and puromycin for selection. BSB8-DRGFP and BSB8-RR-DRGFP cells were then electroporated with increasing concentrations of pCBASceI and pDsRedl-Mito. After five days, cells were harvested and GFP and RFP expressions were detected and quantified as described above. The HR efficiency was established as the number of GFP positive cells with red mitochondrial fluorescence co-localization.

### CRISPR/Cas9 system

BSB8 and BSB8-RR were plated in 6 well plates and transduced with a lentiviral vector encoding Cas9 in presence of polybrene (8 ug/ml) as previously described [30]. At twenty-four hours post-transduction, cells were plated in 100-mm dishes at low density and kept in DMEM complete medium with puromycin for selection. Cas9 expression was induced with doxycycline (2ug/ml) and expression screened by Western blotting. Single cell line clones were selected and screened for Cas9 expression. Cas9 clones were then plated in 6-well tissue culture dishes and transduced with a combination of three lentiviral vectors encoding gRNAs (m1, m2 and m3). At twenty-four hour post-transductions, cells were treated with doxycycline for the induction of Cas9 expression. At 48hrs post-inductions, cells were either harvested for whole cell protein and genomic DNA extraction or re-plated for colony formation assay as described above. The excision of JCV tumor antigens were analyzed by PCR using primers that flank the JCV large T antigen coding region (5′-GCTTATGCCATGCCCTGAAGGT-3′ and 5′-ATGGACAAAGTGCTGAATAGGGA-3′). PCR products were separated on 2% agarose gel by electrophoresis and amplified products were visualized by ethidium bromide staining.
